# Calcium-sensing receptor residues with loss- and gain-of-function mutations are located in regions of conformational change and cause signalling bias

**DOI:** 10.1093/hmg/ddy263

**Published:** 2018-07-20

**Authors:** Caroline M Gorvin, Morten Frost, Tomas Malinauskas, Treena Cranston, Hannah Boon, Christian Siebold, E Yvonne Jones, Fadil M Hannan, Rajesh V Thakker

**Affiliations:** 1 Academic Endocrine Unit, Radcliffe Department of Medicine, Oxford Centre for Diabetes, Endocrinology and Metabolism (OCDEM), University of Oxford, Oxford OX3 7LJ, UK; 2 University of Southern Denmark, Odense C, Denmark; 3 Division of Structural Biology, Wellcome Centre for Human Genetics, University of Oxford, Oxford OX3 7BN, UK; 4 Oxford Molecular Genetics Laboratory, Churchill Hospital, Oxford OX3 7LJ, UK; 5 Institute of Ageing and Chronic Disease, University of Liverpool, Liverpool, UK

## Abstract

The calcium-sensing receptor (CaSR) is a homodimeric G-protein-coupled receptor that signals via intracellular calcium (Ca^2+^_i_) mobilisation and phosphorylation of extracellular signal-regulated kinase 1/2 (ERK) to regulate extracellular calcium (Ca^2+^_e_) homeostasis. The central importance of the CaSR in Ca^2+^_e_ homeostasis has been demonstrated by the identification of loss- or gain-of-function CaSR mutations that lead to familial hypocalciuric hypercalcaemia (FHH) or autosomal dominant hypocalcaemia (ADH), respectively. However, the mechanisms determining whether the CaSR signals via Ca^2+^_i_ or ERK have not been established, and we hypothesised that some CaSR residues, which are the site of both loss- and gain-of-function mutations, may act as molecular switches to direct signalling through these pathways. An analysis of CaSR mutations identified in >300 hypercalcaemic and hypocalcaemic probands revealed five ‘disease-switch’ residues (Gln27, Asn178, Ser657, Ser820 and Thr828) that are affected by FHH and ADH mutations. Functional expression studies using HEK293 cells showed disease-switch residue mutations to commonly display signalling bias. For example, two FHH-associated mutations (p.Asn178Asp and p.Ser820Ala) impaired Ca^2+^_i_ signalling without altering ERK phosphorylation. In contrast, an ADH-associated p.Ser657Cys mutation uncoupled signalling by leading to increased Ca^2+^_i_ mobilization while decreasing ERK phosphorylation. Structural analysis of these five CaSR disease-switch residues together with four reported disease-switch residues revealed these residues to be located at conformationally active regions of the CaSR such as the extracellular dimer interface and transmembrane domain. Thus, our findings indicate that disease-switch residues are located at sites critical for CaSR activation and play a role in mediating signalling bias

## Introduction

**Figure 1:**
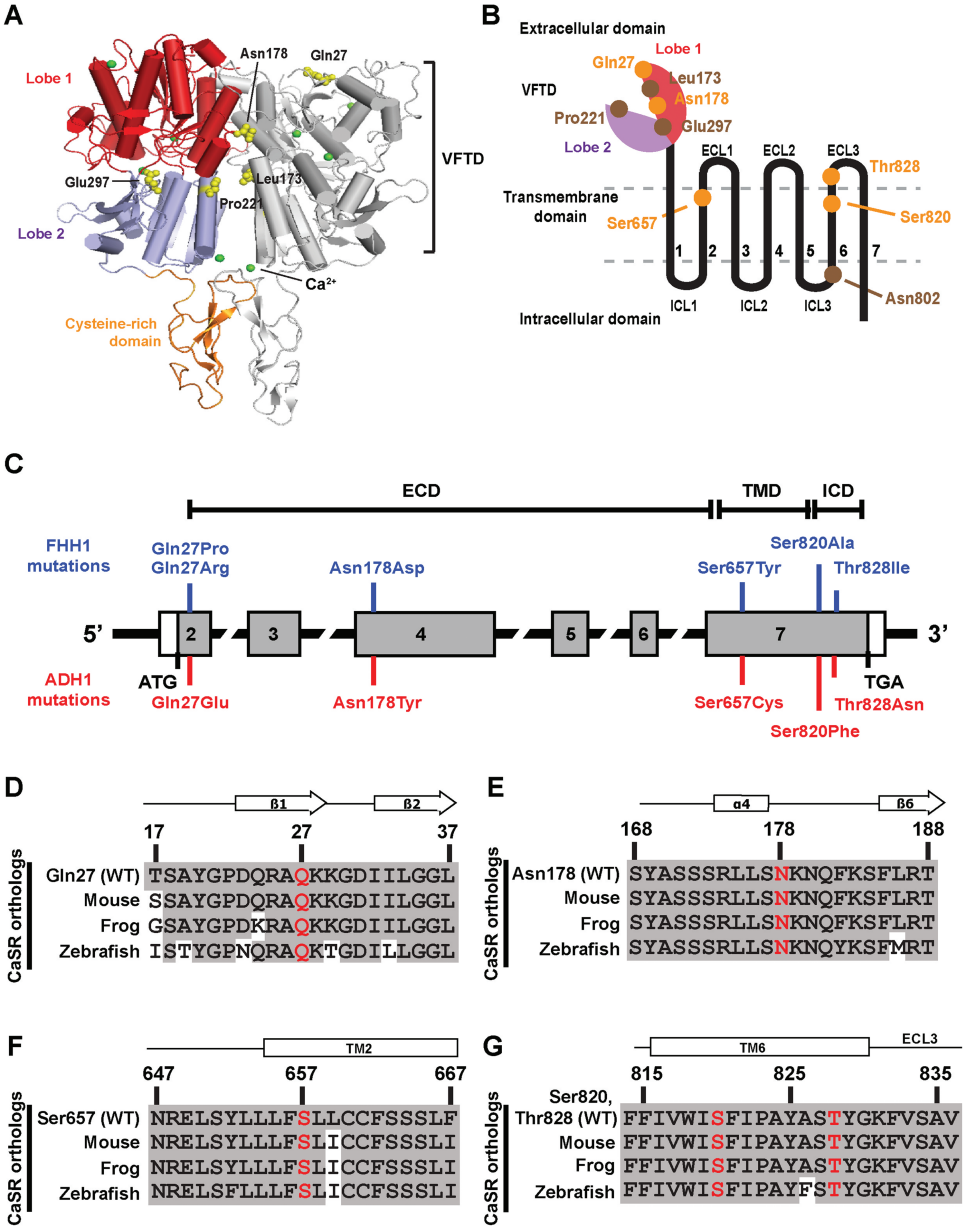
Location and evolutionary conservation of five CaSR disease-switch residues. (**A**) The crystal structure of the homodimeric human CaSR ECD (PDB ID 5K5S) (3). Each CaSR ECD monomer is comprised of a VFTD consisting of two lobes [lobe 1 (red) and lobe 2 (blue)] joined by a hinge region and a cysteine-rich domain (orange). Side chains of disease-switch residues Gln27 and Asn178 and previously reported disease-switch residues Leu173, Pro221 and Glu297 are shown as yellow spheres. Leu173 and Pro221 are located within the hinge region (Fig. S1). The calcium ions are shown as green spheres. (**B**) Schematic diagram of a CaSR monomer showing the extracellular bi-lobed VFTD, seven TMD helices (1–7) with ECL1–3 and ICL1–3 and the ICD. Lobe 1 of the ECD is shown in red and lobe 2 in purple. The locations of the five disease-switch residues (Gln27, Asn178, Ser657, Ser820 and Thr828) are indicated in orange, and reported disease-switch residues are indicated in brown. (**C**) Schematic representation of the genomic organization of the human *CASR* gene showing locations of the FHH1- and ADH1-associated disease-switch residue mutations identified in this study. The *CASR* gene consists of seven exons. Coding regions are shaded grey and untranslated regions are represented by open boxes. The ECD is encoded by exons 1–6 and the 5′ portion of exon 7, and the TMD and ICD by exon 7. The FHH1-associated (blue) and ADH1-associated (red) disease-switch residue mutations are shown above and below the exons, respectively. (**D--G**) Multiple protein sequence alignment of (**D**) residues 17–37 of the ECD β1-strand and β2-strand surrounding Gln27 (Q27); (**E**) residues 168–188 of the ECD α4-helix and β5-strand, surrounding Asn178 (N178) (3,4); (**F**) residues 647–667 of TM helix 2 surrounding Ser657 (S657); and (**G**) residues 815–836 of TM6 and ECL3 surrounding Ser820 (S820) and Thr828 (T828).

The calcium (Ca^2+^)-sensing receptor (CaSR), a member of the class C subfamily of G-protein-coupled receptors (GPCRs), is highly expressed in the parathyroid glands and kidneys and plays an essential role in extracellular calcium (Ca^2+^_e_) homeostasis by regulating parathyroid hormone (PTH) release and urinary Ca^2+^ excretion (1). The CaSR is functionally active as a constitutive homodimer (2), with each monomer of the CaSR consisting of a large extracellular domain (ECD) that has been recently crystallized and shown to comprise a ligand-binding bilobed venus fly-trap domain (VFTD) linked to a cysteine-rich domain (3,4) and also seven transmembrane domains (TMDs) and an intracellular domain (ICD), which are involved in activating downstream signalling proteins (5,6) (Fig. 1A and B). The CaSR couples to two major signal transduction cascades that comprise the G_q/11_-phospholipase C (PLC)-mediated generation of inositol 1,4,5-trisphosphate (IP_3_), which induces a rapid rise in intracellular calcium (Ca^2+^_i_) concentrations (7) and the mitogen-activated protein kinase (MAPK) cascade, such as the extracellular signal-regulated kinase 1/2 (ERK) pathway (8). CaSR-mediated activation of MAPK signalling can occur by coupling to either the G_q/11_ or G_i/o_ pathways (9), and also by a G-protein-independent mechanism involving β-arrestin proteins (10).

The importance of the CaSR for the regulation of Ca^2+^_e_ has been highlighted by the identification of >230 different germline loss- and gain-of-function CaSR mutations that give rise to disorders of Ca^2+^_e_ homeostasis known as familial hypocalciuric hypercalcaemia type 1 (FHH1) and autosomal dominant hypocalcaemia type 1 (ADH1), respectively (11). Structural analysis has shown that FHH1- and ADH1-causing mutations usually affect different CaSR residues with FHH1-causing mutations being scattered throughout the VFTD and TMD regions (11), while ADH1-causing mutations cluster within the second extracellular loop (ECL) of the VFTD (residues 116–136) (12) and also within transmembrane (TM) helices 6 and 7 and the intervening third ECL of the TMD (residues 819–837) (13). However, four CaSR residues have been shown to be the location of both germline loss- and gain-of-function mutations that cause FHH1 and ADH1, respectively, and are termed ‘switch’ or ‘toggle’ residues (4, 14). Three disease-switch residues (Leu173, Pro221 and Glu297) are located within highly conserved regions of the VFTD that are involved in ligand binding (14,15), and one disease-switch residue (Asn802) is located in the third ICL of the TMD (16). The precise role of these disease-switch residues in CaSR function remains to be elucidated, and one possibility is that these residues act as molecular switches, which influence the transitioning of the CaSR between active and inactive conformations and direct CaSR signalling via the Ca^2+^_i_ or MAPK pathways. To gain further insights into the role of CaSR disease-switch residues, we sought for additional residues affected by loss- and gain-of-function mutations, by analysis of familial hypocalciuric hypercalcaemia and autosomal dominant hypocalcaemia patients who had been referred to our centre for diagnostic genetic testing of the *CASR* gene. Furthermore, we determined the effect of disease-switch residue mutations on CaSR-mediated Ca^2+^_i_ and ERK signalling and analysed the structural consequences of these mutations, using the recently determined crystal structures of the CaSR ECD (3,4).

## Results

### Identification of five CaSR disease-switch residues

An analysis of CaSR mutations and variants identified in >300 index cases of FHH1 or ADH1 that had been referred to our centre in Oxford (UK) since 2005 together with a review of previously reported CaSR mutations (17) identified five disease-switch residues, which are the location of both FHH1- and ADH1-associated mutations. Two of these disease-switch residues (Gln27 and Asn178) are located in the CaSR ECD. The Gln27 residue is affected by a novel FHH1-associated p.Gln27Pro variant and a reported FHH1-associated p.Gln27Arg mutation (18), and also a novel ADH1-associated p.Gln27Glu variant (Table 1 and Figs. 1 and S1); whereas the Asn178 residue is affected by a reported FHH1-associated p.Asn178Asp mutation (19) and also by a novel ADH1-associated p.Asn178Tyr variant (Table 1 and Figs. 1 and S1). Two disease-switch residues (Ser657 and Ser820) are located in the CaSR TMD. The Ser657 residue in TM3 is affected by a reported FHH1-associated p.Ser657Tyr mutation (20) and by a novel ADH1-associated p.Ser657Cys variant; whereas the Ser820 residue in TM6 is affected by a novel FHH1-associated p.Ser820Ala variant and a reported ADH1-associated p.Ser820Phe mutation (21) (Table 1 and Fig. 1). One disease-switch residue (Thr828) was shown to be located in ECL3, which links TM6 and TM7 of the CaSR, and this residue is affected by a novel FHH1-associated p.Thr828Ile variant and a novel ADH1-associated p.Thr828Asn variant (Table 1 and Fig. 1). The novel DNA sequence abnormalities identified in this study were absent in >60 700 exomes from the Exome Aggregation Consortium (ExAC) cohort (22) and also affected evolutionary conserved CaSR residues (Fig. 1D–G). Moreover, bioinformatics analyses predicted that these novel variants likely represent pathogenic mutations (Polyphen-2 score, ≥0.90; MutationTasting score, ≥0.99) (Table S1). Thus, this analysis has identified five residues that are sites of both FHH1-associated mutations and ADH1-associated mutations or variants, and these residues may potentially represent molecular switches involved in CaSR activation.

**Table 1:** FHH1- and ADH1-associated mutations affecting five CaSR disease-switch residues

**Wild-type**	**FHH1 mutation**			**ADH1 mutation**		
**Codon** ^**a**^	**Nucleotide**	**Amino acid**	**Reference**	**Nucleotide**	**Amino acid**	**Reference**
**Gln27**	c.80A > C	Pro	*This study*	c.79C > G	Glu	*This study*
		c.80A > G	Arg	(18)		
**Asn178**	c.562A > T	Asp	(19)	c.562A > T	Tyr	*This study*
**Ser657**	c.1970C > A	Tyr	(20)	c.1970C > G	Cys	*This study*
**Ser820**	c.2458 T > G	Ala	*This study*	c.2459C > T	Phe	(21)
**Thr828**	c.2483C > T	Ile	*This study*	c.2483C > A	Asn	*This study*

a ^a^Codon number according to full-length 1078 amino acid CaSR protein. All variants were observed in the heterozygous state.

### Signalling responses of CaSR disease-switch residue mutations

To evaluate the effect of the five FHH1-associated and ADH1-associated ECD and TMD disease-switch residue mutations and variants on CaSR-mediated signalling, HEK293 cells were transiently transfected with pEGFP-N1-CaSR constructs expressing wild-type (WT) or mutant CaSR (Fig. S2), and Ca^2+^_i_ mobilisation and MAPK signalling was assessed following stimulation with Ca^2+^_e_, as reported (10).

#### ECD disease-switch residues.

**Figure 2:**
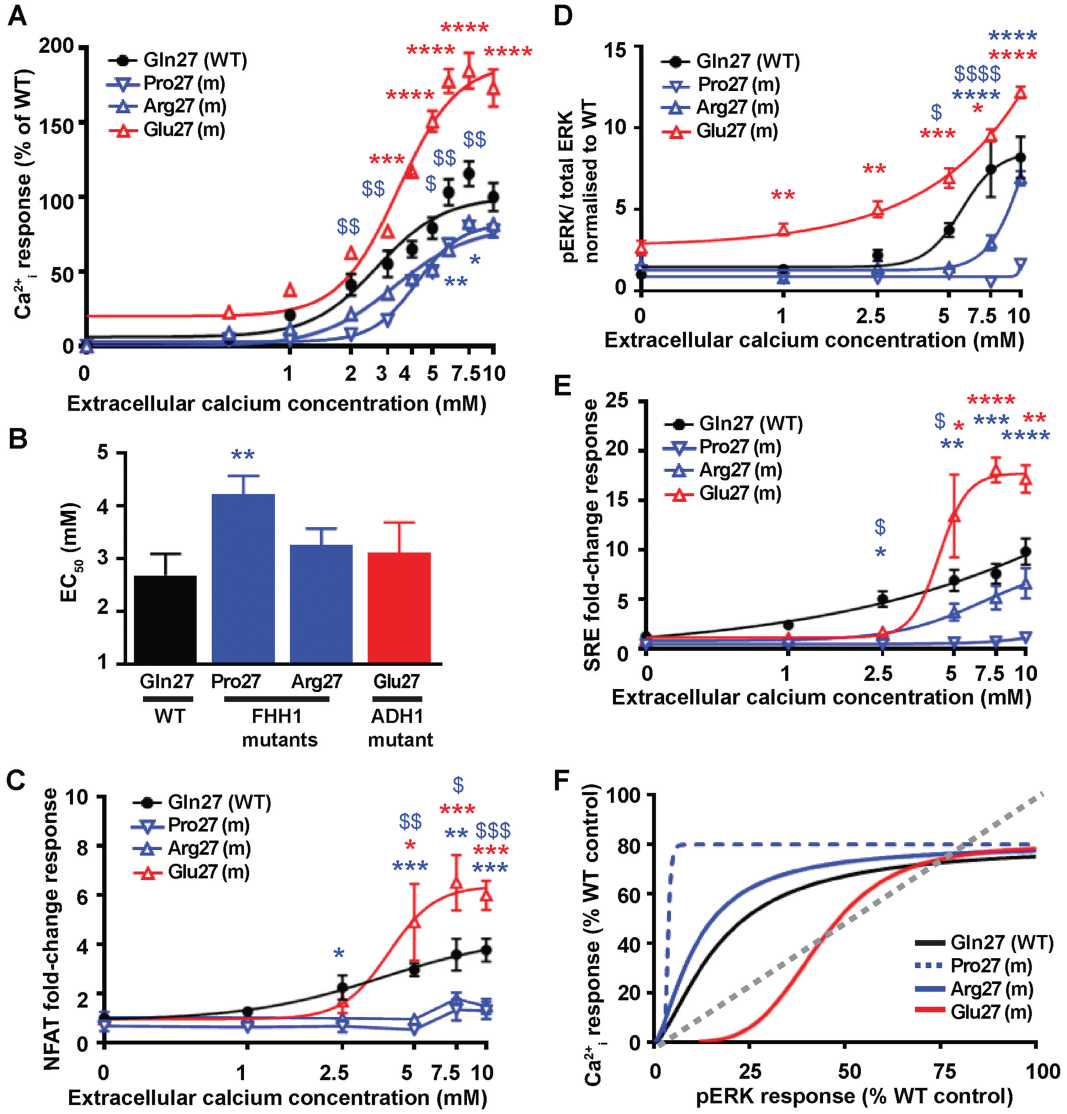
Effect of the ECD Gln27 disease-switch residue mutations on Ca^2+^_i_ and pERK signalling. (**A**) Concentration-response curves showing Ca^2+^_i_ responses following stimulation with Ca^2+^_e_ in HEK293 cells expressing WT (Gln27, black), or FHH1-associated (Pro27 and Arg27, blue) or ADH1-associated (Glu27, red) mutant (m) CaSR proteins. Responses are expressed relative to the WT maximal responses with mean ± SEM of 4–10 biological replicates. (**B**) EC_50_ values obtained from the Ca^2+^_i_ concentration-response curves shown in panel A. (**C–E**) Concentration-response curves of Ca^2+^_e_-induced (**C**) NFAT luciferase reporter responses, (**D**) pERK responses expressed as the ratio of pERK to total ERK concentrations and (**E**) SRE reporter responses of HEK293 cells expressing WT (Gln27) or FHH1-associated (Pro27 and Arg27) or ADH1-associated (Glu27) mutant CaSR proteins. Responses at each Ca^2+^_e_ concentration ([Ca^2+^]_e_) are expressed as a fold-change of basal [Ca^2+^]_e_ responses and shown as mean ± SEM of 6–12 biological replicates. (**F**) Bias plots for Ca^2+^_i_ and pERK signalling responses of mutations affecting the Gln27 CaSR disease-switch residue. Curves located above grey dotted line indicate signalling biased towards Ca^2+^_i_, while curves below grey dotted line indicate signalling biased towards pERK. Statistical analyses comparing WT versus Pro27 (blue dollar) or Arg27 (blue asterisk), and WT versus Glu27 (red asterisk) **** or ^$$$$^*P*-value < 0.0001, or ^$$$^*P*-value < 0.001 ** or ^$$^*P*-value < 0.01, * or ^$^*P*-value < 0.05 compared to WT, by a two-way ANOVA with Tukey’s multiple-comparisons test.

An assessment of Ca^2+^_i_ mobilisation mediated by Gln27 disease-switch residue mutations showed the novel FHH1-associated Pro27 variant protein to cause a rightward shift of the concentration-response curve and led to a significantly increased EC_50_ value compared to WT expressing cells (Fig. 2A and B), consistent with the p.Gln27Pro variant representing a pathogenic loss-of-function CaSR mutation. However, the reported FHH1-associated Arg27 mutant CaSR (18) did not alter the EC_50_ value (Fig. 2B and Table S2), although it did lead to significantly reduced Ca^2+^_i_ responses following stimulation with 5–7.5 mM Ca^2+^_e_ (Fig. 2A). Conversely, cells expressing the novel ADH1-associated Glu27 variant had significantly elevated maximal responses, although EC_50_ values were similar when compared with WT-expressing cells (Fig. 2A and B and Table S2). To further characterise the effects of the Gln27 residue mutations and variants on Ca^2+^_i_ responses, a luciferase reporter containing a response element for nuclear factor of activated T-cells (NFAT), which is commonly used as a downstream mediator of Ca^2+^_i_ signalling (23–25), was utilised. HEK293 cells expressing the FHH1-associated Pro27 and Arg27 CaSR proteins had significantly reduced NFAT fold-change responses compared to WT cells, consistent with a loss-of-function; while the ADH1-associated Glu27 CaSR variant was associated with an elevated NFAT response, consistent with a gain-of-function (Fig. 2C). The effects of the Gln27 residue mutations on MAPK signalling were assessed by measuring fold-change phosphorylated ERK (pERK) responses following exposure to increasing Ca^2+^_e_ concentrations ([Ca^2+^]_e_). Stimulation with Ca^2+^_e_ induced a concentration-dependent increase in pERK responses in WT, Arg27 and Glu27 CaSR-expressing cells (Fig. 2D). These responses were significantly reduced at 5–7.5 mM Ca^2+^_e_ for the FHH1-associated Arg27 mutant compared to WT, whereas cells expressing the FHH1-associated Pro27 mutant had absent pERK responses (Fig. 2D). The ADH1-associated Glu27 mutant had significantly elevated pERK responses at 1–10 mM Ca^2+^_e_, consistent with a gain-of-function in MAPK signalling (Fig. 2D and Table S2). The effects of the Gln27 mutants on MAPK signalling were also assessed by measuring gene transcription induced by a serum-response element (SRE)-containing luciferase reporter construct, which is a downstream mediator of ERK signalling (26,27). These SRE responses were consistent with the pERK responses and indicate that the FHH1-associated Pro27 mutant abolished MAPK signalling (Fig. 2E), and thus exhibits an extreme bias towards Ca^2+^_i_ signalling (Fig. 2F and Table S2); whereas the ADH1-associated Glu27 mutant had significantly elevated SRE responses and showed bias towards the MAPK pathway when compared to WT (Fig. 2F and Table S2).

**Figure 3:**
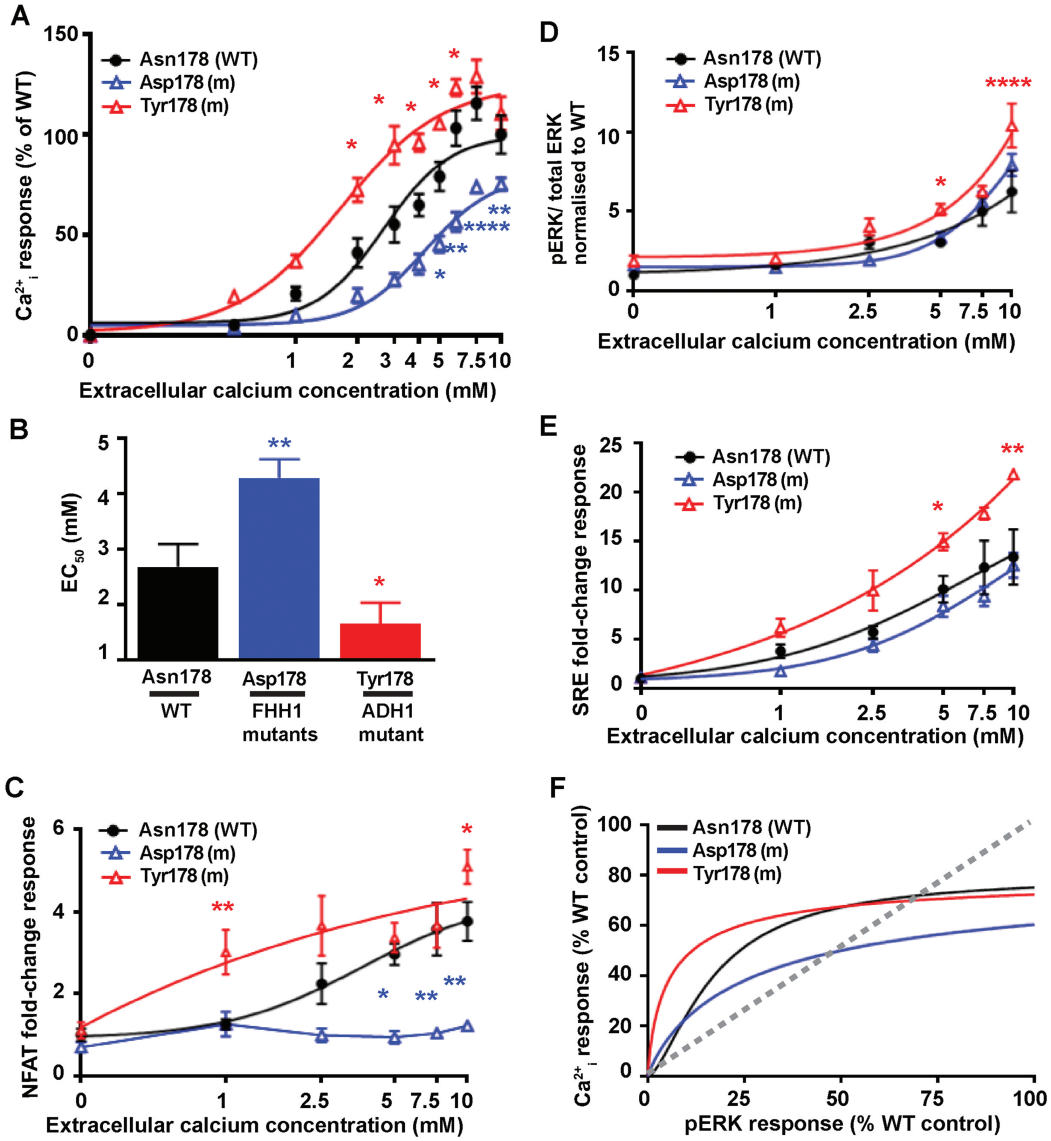
Effect of the ECD Asn178 disease-switch residue mutations on Ca^2+^_i_ and pERK signalling. (**A**) Concentration-response curves showing Ca^2+^_i_ responses following stimulation with Ca^2+^_e_ in HEK293 cells expressing WT (Asn178, black), or FHH1-associated (Asp178, blue) or ADH1-associated (Tyr178, red) mutant (m) CaSR proteins. Responses are expressed relative to the WT maximal responses with mean ± SEM of 4–10 biological replicates. (**B**) EC_50_ values obtained from the Ca^2+^_i_ concentration-response curves shown in panel A. (**C–E**) Concentration-response curves of Ca^2+^_e_-induced (**C**) NFAT luciferase reporter responses, (**D**) pERK responses expressed as the ratio of pERK to total ERK concentrations and (**E**) SRE reporter responses of HEK293 cells expressing WT (Asn178) or FHH1-associated (Asp178) or ADH1-associated (Tyr178) mutant CaSR proteins. Responses at each [Ca^2+^]_e_ are expressed as a fold-change of basal [Ca^2+^]_e_ responses and shown as mean ± SEM of 6–12 biological replicates. (**F**) Bias plots for Ca^2+^_i_ and pERK signalling responses of mutations affecting the Asn178 CaSR disease-switch residue. Curves located above grey dotted line indicate signalling biased towards Ca^2+^_i_, while curves below grey dotted line indicate signalling biased towards pERK. Statistical analyses comparing WT versus Asp178 (blue asterisk), and WT versus Tyr178 (red asterisk) ****P-value < 0.0001, **P-value < 0.01, *P-value < 0.05 compared to WT, by a two-way ANOVA with Tukey’s multiple-comparisons test.

Functional assessment of mutations affecting the Asn178 disease-switch residue showed the reported FHH1-associated Asp178 mutant (19) to cause a rightward shift of the Ca^2+^_i_ concentration-response curve and lead to significantly increased EC_50_ and significantly reduced maximal Ca^2+^_i_ responses (Fig. 3A and B and Table S2). In contrast, cells expressing the novel ADH1-associated Tyr178 variant showed a leftward shift of the concentration-response curve and significantly reduced EC_50_ compared to WT cells (Fig. 3A and B), consistent with the p.Asn178Tyr variant representing a pathogenic gain-of-function CaSR mutation. In keeping with these findings, NFAT reporter responses were also significantly reduced for the FHH1-associated Asp178 mutant and significantly increased for the ADH1-associated Tyr178 mutant (Fig. 3C), thereby indicating that these mutations cause a loss and gain of Ca^2+^_i_ mobilisation, respectively. Evaluation of MAPK signalling showed the ADH1-associated Tyr178 mutant to cause a small but significant increase in pERK responses, whereas the FHH1-associated Asn178 mutant did not impair pERK or SRE reporter responses (Fig. 3D and E). Thus, the loss-of-function Asp178 mutant displayed a bias towards activation of pERK signalling when compared to Ca^2+^_i_ mobilisation, while the gain-of-function Tyr178 mutant favoured signalling by the Ca^2+^_i_ mobilisation pathway, similarly to WT CaSR (Fig. 3F and Table S2).

#### TMD disease-switch residues.

**Figure 4:**
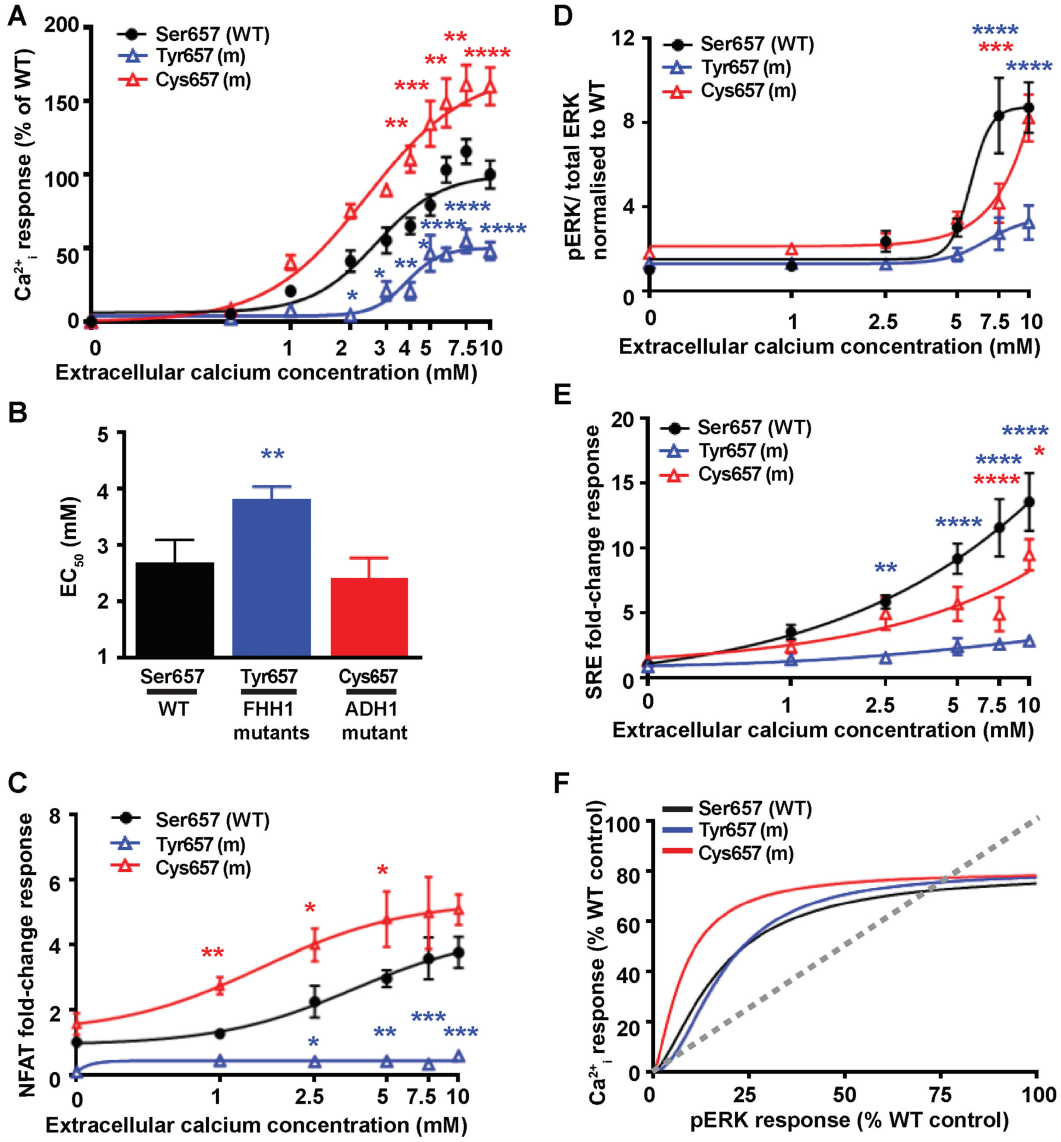
Effect of the TMD Ser657 disease-switch residue mutations on Ca^2+^_i_ and pERK signalling. (**A**) Concentration-response curves showing Ca^2+^_i_ responses following stimulation with Ca^2+^_e_ in HEK293 cells expressing WT (Ser657, black), or FHH1-associated (Tyr657, blue) or ADH1-associated (Cys657, red) mutant (m) CaSR proteins. Responses are expressed relative to the WT maximal responses with mean ± SEM of 4–10 biological replicates. (**B**) EC_50_ values obtained from the Ca^2+^_i_ concentration-response curves shown in panel A. (**C-E**) Concentration-response curves of Ca^2+^_e_-induced (**C**) NFAT luciferase reporter responses, (**D**) pERK responses expressed as the ratio of pERK to total ERK concentrations and (**E**) SRE reporter responses of HEK293 cells expressing WT (Ser657) or FHH1-associated (Tyr657) or ADH1-associated (Cys657) mutant CaSR proteins. Responses at each [Ca^2+^]_e_ are expressed as a fold-change of basal [Ca^2+^]_e_ responses and shown as mean ± SEM of 6–12 biological replicates. (**F**) Bias plots for Ca^2+^_i_ and pERK signalling responses of mutations affecting the Ser657 CaSR disease-switch residue. Curves located above grey dotted line indicate signalling biased towards Ca^2+^_i_, while curves below grey dotted line indicate signalling biased towards pERK. Statistical analyses comparing WT versus Tyr657 (blue asterisk), and WT versus Cys657 (red asterisk) ****P-value < 0.0001, ***P-value < 0.001, **P-value < 0.01, *P-value < 0.05 compared to WT, by a two-way ANOVA with Tukey’s multiple-comparisons test.

An assessment of Ca^2+^_i_ mobilisation mediated by Ser657 disease-switch residue mutations showed the reported FHH1-associated Tyr657 mutant (20) to cause significantly decreased Ca^2+^_e_-mediated Ca^2+^_i_ maximal responses, and a significantly elevated Ca^2+^_i_ EC_50_ value compared to WT (Fig. 4A and B and Table S2). The novel ADH1-associated Cys657 variant led to significantly increased maximal Ca^2+^_i_ responses, although the Ca^2+^_i_ EC_50_ response of the Cys657 protein was not significantly altered compared to WT (Fig. 4A and B and Table S2). To further characterise the functional consequences of the Ser657 residue mutations and variants, Ca^2+^_i_ responses were determined using the NFAT reporter assay. This showed the FHH1-associated Tyr657 mutant to have abolished NFAT reporter responses, whereas the novel ADH1-associated Cys657 variant displayed significantly increased NFAT reporter activity (Fig. 4C), consistent with this representing a pathogenic gain-of-function CaSR mutation. An assessment of MAPK signalling showed the FHH1-associated Tyr657 mutant to have significantly reduced pERK and SRE reporter responses (Fig. 4D and E) consistent with this mutation leading to CaSR inactivation. In contrast, the ADH1-associated Cys657 mutant did not alter pERK or SRE reporter responses unless stimulated with supraphysiological (≥7.5 mM) [Ca^2+^_e_], which caused this mutant to have significantly impaired MAPK signalling (Fig. 4D and E). Thus, the FHH1-associated Tyr657 and ADH1-associated Cys657 mutant CaSR proteins result in biased signalling towards the Ca^2+^_i_ pathway (Fig. 4F and Table S2).

**Figure 5:**
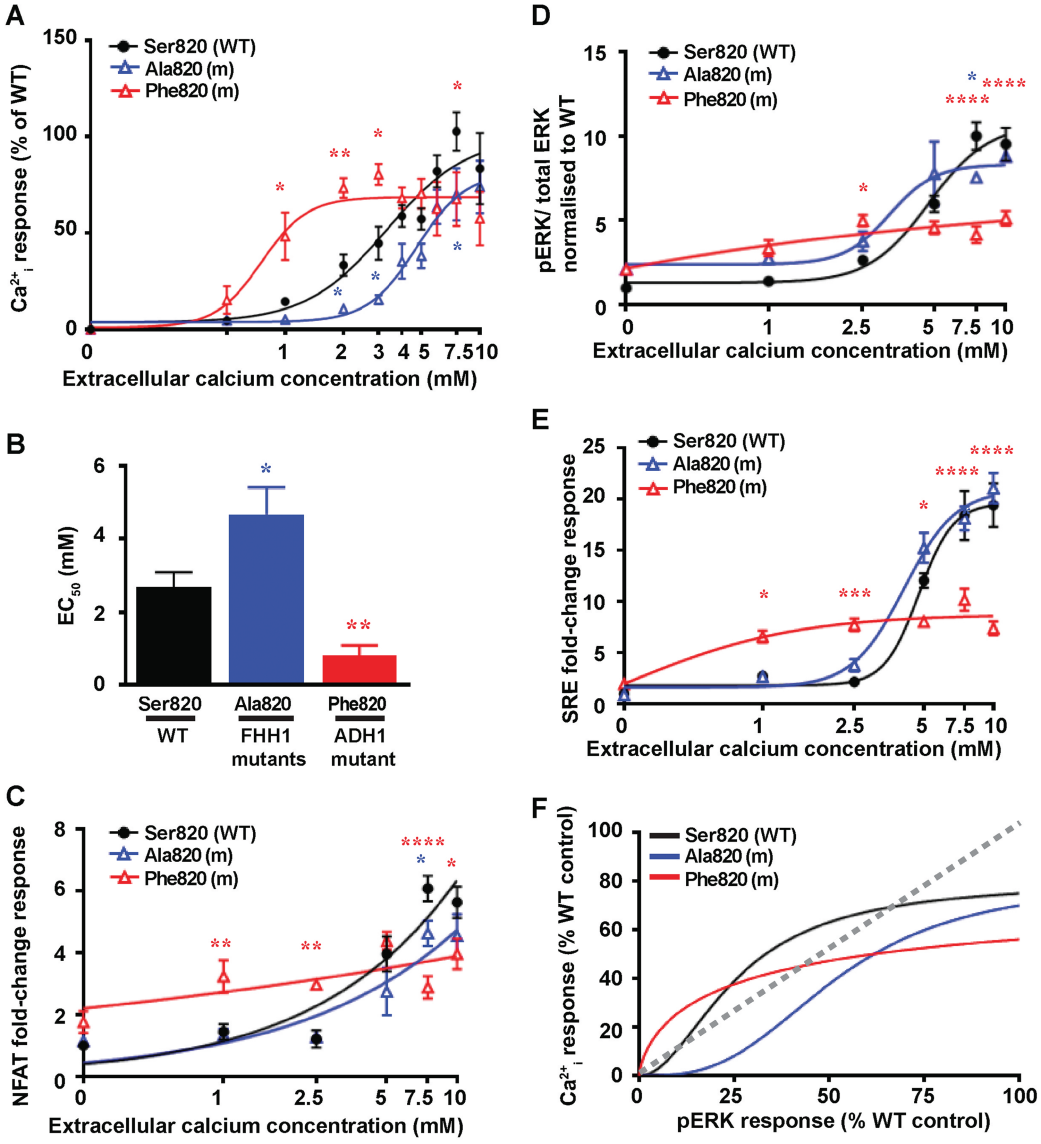
Effect of the TMD Ser820 disease-switch residue mutations on Ca^2+^_i_ and pERK signalling. Concentration-response curves showing Ca^2+^_i_ responses following stimulation with Ca^2+^_e_ in HEK293 cells expressing WT (Ser820, black) or FHH1-associated (Ala820, blue) or ADH1-associated (Phe820, red) mutant (m) CaSR proteins. Responses are expressed relative to the WT maximal responses with mean ± SEM of 4–10 biological replicates. (**B**) EC_50_ values obtained from the Ca^2+^_i_ concentration-response curves shown in panel A. (**C–E**) Concentration-response curves of Ca^2+^_e_-induced (**C**) NFAT luciferase reporter responses, (**D**) pERK responses expressed as the ratio of pERK to total ERK concentrations and (**E**) SRE reporter responses of HEK293 cells expressing WT (Ser820),or FHH1-associated (Ala820) or ADH1-associated (Phe820) mutant CaSR proteins. Responses at each [Ca^2+^]_e_ are expressed as a fold-change of basal [Ca^2+^]_e_ responses and shown as mean ± SEM of 6–12 biological replicates. (**F**) Bias plots for Ca^2+^_i_ and pERK signalling responses of mutations affecting the Ser820 CaSR disease-switch residue. Curves located above grey dotted line indicate signalling biased towards Ca^2+^_i_, while curves below grey dotted line indicate signalling biased towards pERK. Statistical analyses comparing WT versus Ala820 (blue asterisk), and WT versus Phe820 (red asterisk) ****P-value < 0.0001, **P-value < 0.01, *P-value < 0.05 compared to WT, by a two-way ANOVA with Tukey’s multiple-comparisons test.

Functional assessment of mutations affecting the Ser820 disease-switch residue showed the novel FHH1-associated Ala820 variant to cause a rightward shift of the concentration-response curve and significantly increased EC_50_ value (Fig. 5Aand B and Table S2), which indicated that this represents a pathogenic loss-of-function CaSR mutation. Whereas, the reported ADH1-associated Phe820 mutant (21) showed a significantly reduced EC_50_ value (Fig. 5A and B and Table S2), consistent with a gain of CaSR function. In keeping with these findings, the ADH1-associated Phe820 mutant showed significantly elevated NFAT reporter responses at 1–2.5 mM [Ca^2+^]_e_, while the FHH1-associated Ala820 mutant showed reduced NFAT reporter responses at 7.5 mM [Ca^2+^]_e_ (Fig. 5C). Assessment of MAPK responses revealed that the FHH1-associated Ala820 mutant induced a modest reduction in pERK responses following stimulation with 7.5 mM [Ca^2+^]_e_ and had no effect on SRE reporter responses when compared to WT (Fig. 5D and E). The ADH1-associated Phe820 mutant showed a bimodal effect on MAPK signalling, with a significant increase in pERK and SRE responses at low [Ca^2+^]_e_ (1–2.5 mM), but reduced responses at high [Ca^2+^]_e_ (5-10 mM) compared to WT (Fig. 5D and E). Bias plots demonstrated that the FHH1-associated Ala820 mutant was biased towards MAPK signalling, while the Phe820 cells was more biased towards Ca^2+^_i_ signalling (Fig. 5F and Table S2).

#### ECL3 disease-switch residue.

**Figure 6:**
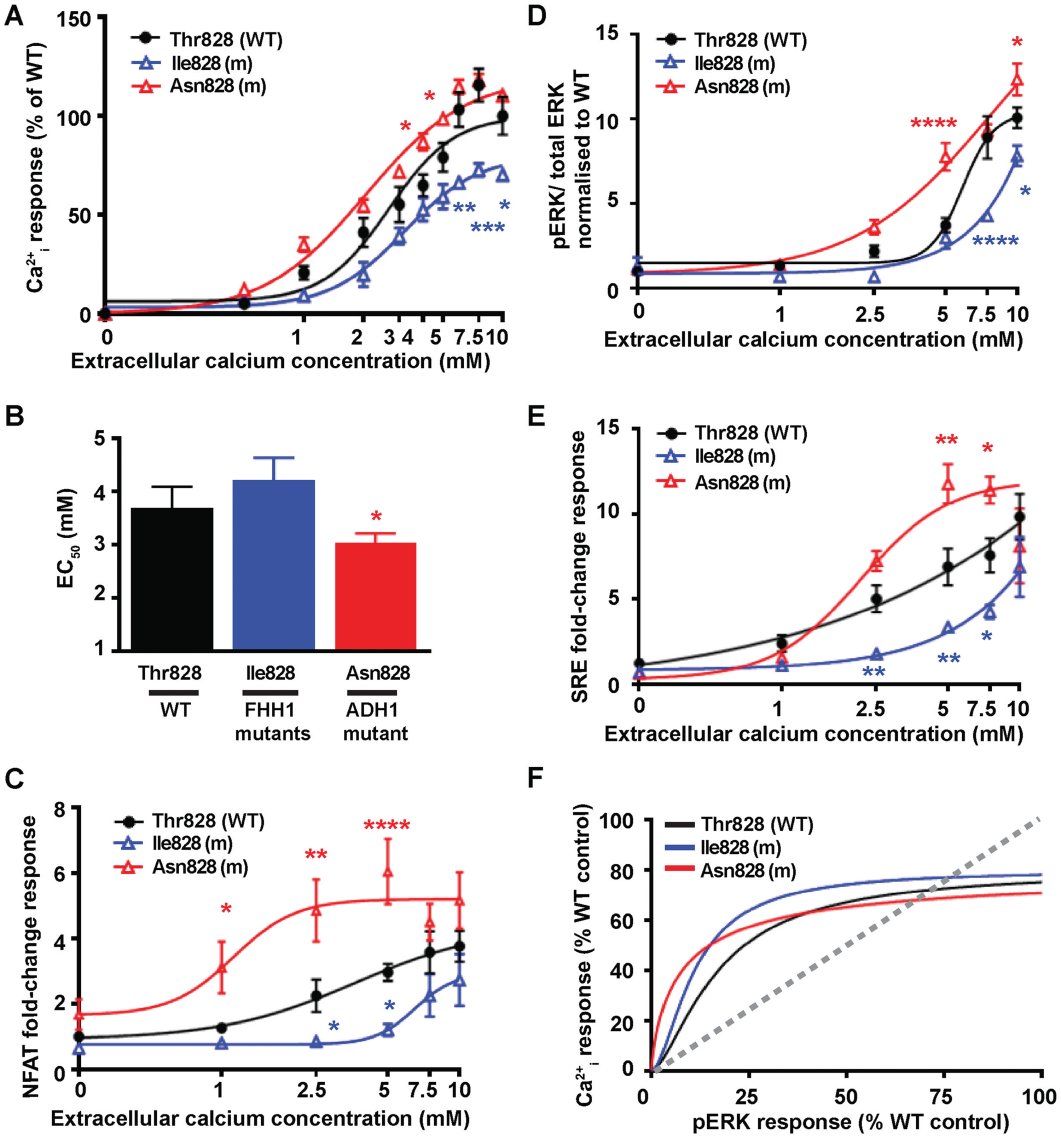
Effect of the TMD Thr828 disease-switch residue mutations on Ca^2+^_i_ and pERK signalling. Concentration-response curves showing Ca^2+^_i_ responses following stimulation with Ca^2+^_e_ in HEK293 cells expressing WT (Thr828, black) or FHH1-associated (Ile828, blue) or ADH1-associated (Asn828, red) mutant (m) CaSR proteins. Responses are expressed relative to the WT maximal responses with mean ± SEM of 4–10 biological replicates. (**B**) EC_50_ values obtained from the Ca^2+^_i_ concentration-response curves shown in panel A. (**C–E**) Concentration-response curves of Ca^2+^_e_-induced (**C**) NFAT luciferase reporter responses, (**D**) pERK responses expressed as the ratio of pERK to total ERK concentrations and (**E**) SRE reporter responses of HEK293 cells expressing WT (Thr828) or FHH1-associated (Ile828) or ADH1-associated (Asn828) mutant CaSR proteins. Responses at each [Ca^2+^]_e_ are expressed as a fold-change of basal [Ca^2+^]_e_ responses and shown as mean ± SEM of 6–12 biological replicates. (**F**) Bias plots for Ca^2+^_i_ and pERK signalling responses of mutations affecting the Thr828 CaSR disease-switch residue. Curves located above grey dotted line indicate signalling biased towards Ca^2+^_i_ while curves below grey dotted line indicate signalling biased towards pERK. Statistical analyses comparing WT versus Ile828 (blue asterisk), and WT versus Asn828 (red asterisk) ****P-value < 0.0001, ***P-value < 0.001, **P-value < 0.01, *P-value < 0.05 compared to WT, by a two-way ANOVA with Tukey’s multiple-comparisons test.

Functional assessment of mutations affecting the Thr828 disease-switch residue showed the novel FHH1-associated Ile828 variant to cause significantly decreased Ca^2+^_e_-mediated Ca^2+^_i_ maximal responses, while the novel ADH1-associated Asn828 variant led to significantly increased Ca^2+^_i_ responses and reduced EC_50_ value compared to WT (Fig. 6A and B and Table S2). To further characterise the functional consequences of the Thr828 residue variants, Ca^2+^_i_ responses were determined using the NFAT reporter assay. This showed the FHH1-associated Ile828 variant to have significantly reduced NFAT responses, whereas the ADH1-associated Asn828 variant had significantly increased NFAT responses (Fig. 6C), consistent with these variants representing pathogenic loss- and gain-of-function CaSR mutations, respectively. In keeping with the findings of the Ca^2+^_i_ signalling studies (Fig. 6A–C), an assessment of MAPK signalling showed the FHH1-associated Ile828 mutation to have significantly reduced pERK and SRE responses, whereas the ADH1-associated Asn828 mutation had significantly increased MAPK responses (Fig. 6D and E). Bias plots showed that both FHH1 and ADH1 mutants at the 828 residue had similar bias towards the Ca^2+^_i_ pathway to WT expressing cells (Fig. 6F).

### Cellular expression of CaSR disease-switch residue mutations

Western blot analysis of whole cell lysates and plasma membrane fractions of HEK293 cells transiently transfected with WT or mutant CaSR disease-switch residue proteins was undertaken to determine whether alterations in cellular expression may have contributed to the loss- and gain-of-function caused by these mutant proteins. Untransfected HEK293 cells were shown not to endogenously express CaSR protein (Fig. S3A), whereas transfected cells expressed the monomeric CaSR as two bands at 140 and 160 kDa, and the dimeric CaSR at ~250 kDa (Fig. S3A), consistent with previous reports (28). An assessment of the FHH1-associated and ADH1-associated ECD Gln27 and Asn178 disease-switch residue mutations showed no alterations in the total cellular or cell-surface expression of the monomeric and dimeric forms of the CaSR compared to WT (Fig. S3B and C). In contrast, reduced total and cell surface expression was observed for the FHH1-associated TMD Tyr657 mutant protein (Fig. S3D), as reported (29). However, no alterations in expression were noted for mutations affecting the TMD Ser820 and Thr828 CaSR residues (Fig. S3E and F).

### Structural characterisation of the CaSR disease-switch residues

**Figure 7:**
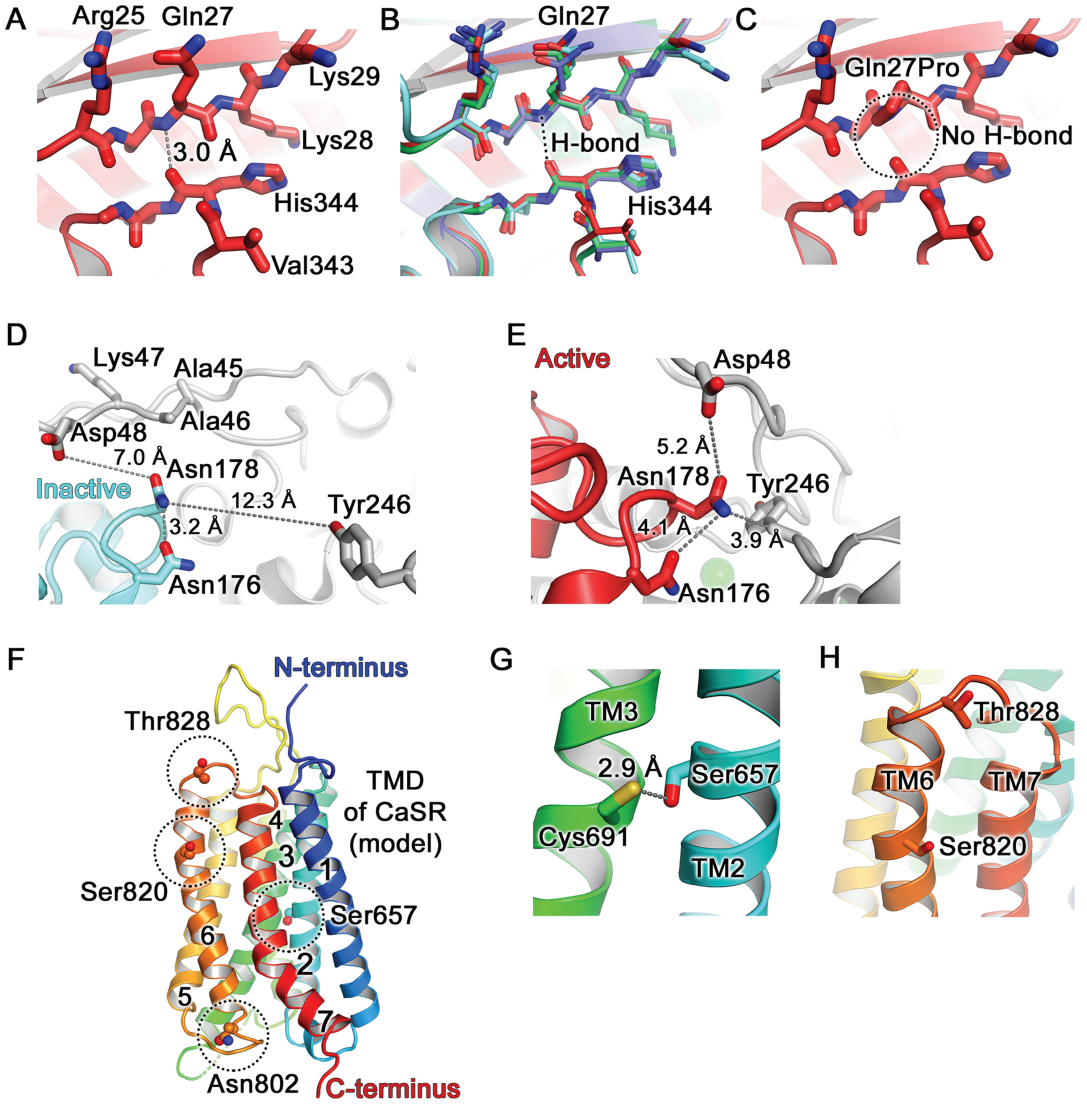
Structural analysis of CaSR disease-switch residues**.** (**A**) The Gln27 residue is located in the β1-strand of lobe 1 of the CaSR ECD, shown in the active conformation (PDB: 5K5S) (3) (Figs. 1 and S1). The peptide backbone amino group hydrogen on nitrogen (blue) of Gln27 forms a hydrogen bond (H-bond) with the peptide carbonyl group of His344 located in the α10 and α11 loop. (**B**) Superposition of all known crystal structures of CaSR: active conformation (carbons are in dark red as in panels A and C; PDB: 5K5S) (3), inactive conformation (cyan, PDB: 5K5T) (3), cyclomethyltryptophan/magnesium-bound form (green, PDB: 5FBK) (4), cyclomethyltryptophan/magnesium/gadolinium-bound form (blue, PDB: 5FBH,) (4). The Gln27-His344 interaction is not altered in the active and inactive conformations of the CaSR. (**C**) The FHH1-associated p.Gln27Pro mutation abolishes the Gln27-His344 hydrogen bond (circled). (**D**) The Asn178 is located at the lobe 1–lobe 1 dimer interface (Figs. 1 and S1). The side chain of Asn178 is exposed to solvent in the inactive conformation (cyan, PDB: 5K5T (3)) and is positioned at ≥7.0 Å from neighbouring residues (Asp48, Tyr246) on the opposing CaSR molecule. (**E**) The side chain of Asn178 is located within a tightly packed environment between two CaSR ECD protomers in the active conformation (PDB: 5K5S). Mutation to Asp178 or Tyr178 could alter the relative orientation between the two protomers. (**F**) Homology model of the CaSR TMD viewed parallel to the cell membrane and based on the structure of mGluR1 (PDB: 4OR2) (30). Side chains of Ser657, Asn802, Ser820 and Thr828 are shown as spheres (dashed circles). Seven TM helices are numbered. (**G**) The Ser657 residue is located within TM2 and its side chain projects towards Cys691 located on TM3. (**H**) The Ser820 residue is located within TM6 and its side chain points towards the transmembrane bilayer. The Thr828 residue is located within ECL3, which connects TM6 and TM7.

The reported crystal structures of the ligand- and non-ligand-bound forms of the homodimeric form of the bilobed CaSR ECD (3,4) were analysed to determine whether the ECD disease-switch residues (Gln27 and Asn178) may act as molecular switches that could influence the adoption of the CaSR into an active or inactive conformation (Fig. 7A–E). Such crystal structures for the CaSR TMD are not established, and the structural properties of the TMD and ECL3 disease-switch residues (Ser657, Ser820 and Thr828) were therefore characterised using a homology model of the CaSR TMD (Fig. 7F–H), that is based on the crystal structure of human metabotropic glutamate receptor type 1 (mGluR1) in the inactive state, as previously described (10, 30). The location of the four previously reported CaSR disease-switch residues (Leu173, Pro221, Glu297 and Asn802) (14–16) were also mapped onto the ECD and TMD of the CaSR (Figs. 1A and B, 7F and S1).

#### ECD disease-switch residues.

The Gln27 disease-switch residue is located within lobe 1 of the VFTD and forms hydrogen bonds with the adjacent His344 (Fig. 7A–C). The side chain of Gln27 adopts similar conformations in all crystal structures (Fig. 7B), and the FHH1-associated p.Gln27Pro (Fig. 7B and C) mutation is predicted to abolish a hydrogen bond between an amino group of Gln27 and carbonyl oxygen of His344 (Fig. 7B and C). A similar analysis of the FHH1-associated p.Gln27Arg and ADH1-associated p.Gln27Glu mutations did not reveal the occurrence of any structural alterations involving the VFTD, using the available crystal structures (3,4). The Asn178 disease-switch residue is located at the lobe 1–lobe 1 VFTD dimer interface (Figs. 1, 7D and E) and, in the inactive conformation (Fig. 7D), forms an intramolecular hydrogen bond with the neighbouring Asn176 residue. However, in the active (ligand-bound) conformation (Fig. 7E), Asn178 could participate in hydrophilic interactions with the neighbouring Asn176 residue and form hydrogen bond interactions with Tyr246 from the opposing molecule in the CaSR dimer, thereby stabilising an active conformation of CaSR (3). The FHH1-associated p.Asn178Asp mutation is predicted to introduce a negative charge at the dimer interface, which could lead to repulsion between Asp178 and Asp48 from the opposing molecule in the active conformation (Fig. 7E). Thus, the p.Asn178Asp mutation may impair dimerisation and destabilise the active CaSR conformation. In contrast, the ADH1-associated p. Asn178Tyr mutation is predicted to introduce a bulky residue at the dimer interface that may potentially form pi–pi interactions with Tyr246 from the opposing molecule and stabilise the CaSR dimer (Fig. 7E), thereby leading to CaSR activation. Structural analysis also showed that the reported Leu173 (lobe 1) and Pro221 (lobe 2) disease-switch residues (14) are located at the hinge region of the VFTD dimer interface, while the reported Glu297 disease-switch residue (15) is located within the VFTD cleft which is involved in ligand binding (Figs. 1A and S1) (3).

#### TMD and ECL3 disease-switch residues.

The Ser657 disease- switch residue is located in the middle of TM2 (Fig. 7F) and its side chain projects towards Cys691 of TM3 (Fig. 7G). The FHH1-associated p.Ser657Tyr mutation would introduce a bulky side chain, which may disrupt the TM2–TM3 interactions. In contrast, the ADH1-associated p.Ser657Cys mutation would place two Cys residues in close proximity, and this may lead to the formation of a disulphide bond between TM2 and TM3 (Fig. 7G) that could potentially stabilise the CaSR in an active conformation. The Ser820 disease-switch residue is located in the middle of TM6, while Thr828 is in ECL3, which connects TM6 with TM7 (Fig. 7F and H). The reported Asn802 disease-switch residue (16) is also located in the distal TMD and is situated in ICL3, which connects TM5 with TM6 (Fig. 7F). Homology model analysis did not predict any interactions between these distal TMD disease-switch residues and neighbouring amino acid residues. However, these residues (Ser657, Asn802, Ser820 and Thr828) are located within or adjacent to TM3 and TM6, which can undergo large conformational changes important for G-protein binding (31,32) and thus these disease-switch residues may potentially affect G-protein activation without influencing interactions with neighbouring residues.

## Discussion

Our studies have identified five CaSR disease-switch residues, and these findings together with the four previously reported disease-switch residues (14–16) indicate that the CaSR has at least nine disease-switch residues, which are the location of both germline loss- and gain-of-function mutations that lead to FHH1 and ADH1, respectively (Fig. 1). The finding that different mutations affecting the same residue may lead to distinct genetic disorders agrees with previous studies of the human vasopressin type 2 receptor (V2R), which is a class A GPCR involved in the regulation of renal water excretion (33,34). Thus, a loss-of-function germline mutation affecting the highly conserved Arg137 residue of the V2R, which is located in the TMD, has been shown to cause nephrogenic diabetes insipidus, which is characterized by excess renal water loss (33), whereas gain-of-function mutations of the V2R Arg137 residue give rise to inappropriate renal water retention that causes the nephrogenic syndrome of inappropriate antidiuresis (NSIAD) (34).

Structural analysis of the CaSR demonstrated that four extracellular disease-switch residues are located in key sites within the VFTD (Figs. S1 and 7). Thus, the reported Glu297 disease-switch residue (15) is located within the cleft formed by lobes 1 and 2 of the VFTD (Fig. 1) and has been shown to be involved in ligand binding which in turn leads to CaSR activation by mediating closure of the two lobes of the VFTD (3). Whereas, the Leu173, Asn178 and Pro221 disease-switch residues are located in the proximity of the hinge region (Figs. 1A and S1) between lobes 1 and 2, and also at the extended dimer interface that is formed by the two protomers of the VFTD upon agonist-mediated CaSR activation (Fig. 1) (3). Moreover, three of the TMD disease-switch residues clustered around TM6 (Figs. 1 and 7), which has been shown in structural studies of class A and B GPCRs to undergo a substantial outward movement in order to accommodate G-protein binding during receptor activation (35–37). Our finding that ECD and TMD CaSR disease-switch residues are located within regions that undergo large conformational changes upon ligand binding is consistent with an analysis of molecular switch residues identified in class A GPCRs, which has shown these to be located between the GPCR ligand-binding pocket and the G-protein interaction site (38). In this region of the TMD, class A GPCR molecular switch residues were shown to particularly cluster around the kink region of TM6, which undergoes the greatest structural alteration during receptor activation (38). Moreover, large conformational changes upon ligand binding involving residues within TM3 and TM6 have also been shown to be important in class C GPCR activation and G-protein coupling (39,40).

We used CaSR crystal structures (3,4) to obtain detailed insights into how the two VFTD disease-switch residues iden-tified in this study may be influencing CaSR activation. Our analysis indicated that the Asn178 residue adopts distinct con-formations and forms different interactions in the ligand bound and unbound forms of the CaSR (Fig. 7D and E). In the inactive CaSR, Asn178 forms weak intramolecular hydrogen bonds with Asn176, whereas upon ligand binding Asn178 interacts with the opposing molecule and could facilitate agonist-induced dimerisation of the VFTD, which in turn triggers CaSR-mediated signal transduction (3). It is therefore possible that the Asn178 residue and its mutations may switch the CaSR between active and inactive conformations. This finding that residues affected by loss- and gain-of-function mutations can act as molecular switches is consistent with that previously reported for the Arg137 molecular switch residue of the human V2R, which participates in an ionic lock mechanism common to class A GPCRs and determines whether these receptors adopt active or inactive conformations (33,34). However, our analysis of the Gln27 residue within the context of the available crystal structures revealed that this residue was unlikely to be acting as a molecular switch as its interactions with other residues did not change upon CaSR activation (Fig. 7B). Moreover, Gln27 is not located in a region directly involved in ligand-binding or in mediating large conformational changes. Instead, the finding that Gln27 mutations can lead to loss- or gain-of-function is most likely a consequence of the specific mutations affecting this residue rather than due to the properties of the WT residue. Detailed structural analysis of the TMD disease-switch residues was not possible because the crystal structure of the CaSR TMD has not been established. However, homology modelling of the CaSR TMD based on the crystal structure of the related mGluR1 (10) provided insights into the role of the Ser657 disease-switch residue, which may mediate interactions between TM2 and TM3 (Fig. 7G). It should be noted that all of the TMD disease-switch residues detected to-date (Ser657, Asn802, Ser820 and Thr828) have polar side chains. Previous studies of class A GPCRs have shown that conserved TMD polar residues contribute to a water-mediated hydrogen-bonded network within the TMD that plays a key role in regulating receptor activity and that engineered alanine substitutions of these residues can cause loss or gain of receptor function (41). Thus, the polar TMD disease-switch residues may potentially play a complex role in mediating interactions between the different helices within the CaSR TMD.

*In vitro* functional expression studies of mutations affecting the five disease-switch residues identified in this study have provided insights into the role of these residues in mediating CaSR signal transduction. For example, most of the disease-switch residue mutations showed similar levels of cell-surface expression compared to WT CaSR (Fig. S3), and it is thus unlikely that the alterations in signalling caused by these mutants is a consequence of altered anterograde or retrograde receptor trafficking. Moreover, none of the disease-switch residue mutations had significant effects on CaSR function in the absence of the agonist (Ca^2+^_e_), and therefore these disease-switch residues are not involved in regulating the basal activity of the CaSR. However, mutations affecting the Gln27, Ser657 and Thr828 disease-switch residues altered the maximal responses of the CaSR without influencing EC_50_ values, which may indicate that these mutations altered the intrinsic signalling efficacy of the CaSR without affecting agonist affinity (42). Another possibility is that the alterations in maximal Ca^2+^_i_ responses may be due to changes in cell-surface expression of the CaSR switch residue mutants. However, an alteration in cell-surface expression was only demonstrated for the Tyr657 disease-switch residue mutant. Moreover, in contrast with other ADH1-associated disease-switch residue mutants, which showed increased maximal Ca^2+^_i_ responses, the gain-of-function Phe820 disease-switch residue mutant showed reduced maximal Ca^2+^_i_ responses compared to WT, which indicates that the CaSR activation caused by this mutant may have led to depletion of intracellular stores of Ca^2+^, as reported (29).

As most of the nine CaSR disease-switch residues are located in regions of the CaSR that are predicted to undergo conformational rearrangements upon ligand binding (Figs. 1 and 7) (3), mutations affecting these residues have the potential to stabilise the CaSR in conformations that selectively activate G-protein dependent or independent signalling pathways (43). In keeping with this, many of the CaSR disease-switch residue mutations showed distinct patterns of signalling bias compared to the WT CaSR, which preferentially activates Ca^2+^_i_ mobilisation (29). The disease-switch residue mutations were commonly found to show signalling bias towards either Ca^2+^_i_ (FHH1-associated p.Gln27Pro or ADH1-associated p.Asn178Tyr and p.Ser657Cys) or MAPK pathway activation (FHH1-associated p.Asn178Asp and p.Ser820Ala, or ADH1-associated p.Gln27Glu). In particular, the FHH1-associated p.Asn178Asp and p.Ser820Ala mutations led to reductions in Ca^2+^_i_ mobilisation without any significant impairment of pERK responses. Thus, these mutations may result in the CaSR adopting conformational states that favour signalling away from G_q/11_-mediated PLC activation and towards G_i/o_ and/or β-arrestin-mediated MAPK signalling, as has been recently demonstrated for a CaSR mutation that disrupts a salt-bridge within the TMD (10). In contrast, the ADH1-associated p.Ser657Cys disease-switch residue mutation showed signalling bias towards Ca^2+^_i_ mobilization, as this mutation significantly increased Ca^2+^_i_ responses, yet led to a reduction in MAPK signalling as assessed by pERK and SRE reporter responses (Fig. 4). To our knowledge, this is the first disease-causing CaSR mutation reported to cause both loss- and gain-of-function, depending on which signalling pathway is evaluated, and highlights a potential uncoupling of Ca^2+^_i_ and MAPK responses. This understanding of signalling bias caused by CaSR mutations may provide a precision medicine approach to the treatment of patients with symptomatic forms of FHH1 and ADH1. For example, FHH1 patients harbouring a CaSR mutation such as p.Ser820Ala, which shows a preferential impairment of Ca^2+^_i_ responses, may benefit from treatment with cinacalcet, as this CaSR positive allosteric modulator (PAM) is biased towards the Ca^2+^_i_ mobilisation pathway (44), whereas FHH1-causing mutations such as p.Thr828Ile that cause a preferential reduction in MAPK signalling may benefit from treatment with the AC265347 compound, as this CaSR PAM causes biased activation of pERK (45).

In conclusion, our studies have demonstrated that CaSR residues affected by disease-causing loss- and-gain-of-function mutations are located within receptor domains that are pivotal for CaSR function and may undergo conformational rearrangements during GPCR activation. Moreover, these disease-switch residues likely determine whether the CaSR signals via Ca^2+^_i_ or MAPK pathways.

## Materials and Methods

### Bioinformatic analysis of DNA sequence variants

Publicly accessible databases [dbSNP (http://www.ncbi.nlm.nih.gov/projects/SNP/) (46) and the ExAC (exac.broadinstitute.org), which contains details from exomes of 60 706 unrelated individuals (22)] were examined for the presence of *CASR* sequence variants.

### Protein sequence alignment and structural analysis

Protein sequences of CaSR orthologs were aligned using ClustalOmega (http://www.ebi.ac.uk/Tools/msa/clustalo/) (47). Crystal structures of CaSR ECDs in both active and inactive conformations were analysed [Protein Data Bank (PDB) accession codes 5FBK, 5FBH, 5K5S, 5K5T] (3,4). The HHpred homology detection server (https://toolkit.tuebingen.mpg.de/hhpred) was used to identify crystal structures with similar sequences to the CaSR TMD, and to perform amino acid sequence alignment (30,48). The CaSR sequence was threaded onto the mGluR1 (PDB ID 4OR2) (30) template coordinates using Modeller (https://toolkit.tuebingen.mpg.de/modeller) to construct a homology model (49). Figures were prepared using the PyMOL Molecular Graphics System (Schrodinger, LLC).

### Cell culture and transfection

Studies were performed in HEK293 cells maintained in DMEM-Glutamax media (ThermoFisher) with 10% fetal bovine serum (Gibco) at 37°C, 5% CO_2_. Mutations were introduced into the reported WT pEGFP-N1-CaSR construct by site-directed mutagenesis using the Quikchange Lightning Kit (Agilent Technologies) and gene-specific primers (SigmaAldrich), as described. WT and mutant pEGFP-N1-CaSR constructs, and luciferase reporter constructs (pGL4.30-NFAT and pGL4.33-SRE, Promega) were transiently transfected into HEK293 cells using Lipofectamine 2000 (LifeTechnologies) 48 h before experiments, as described (50).

### Western blot analyses

Expression of WT and mutant proteins by the pEGFP-N1-CaSR constructs was confirmed by Western blot analysis, with the calnexin housekeeping protein being used as a loading control (50). For cell fractionation studies, cells were transfected with CaSR constructs and 48 h later plasma membrane and cytoplasmic fractions were isolated using a plasma membrane extraction kit (Catalog No. 65400, Abcam), as described (51). Plasma membrane calcium ATPase (PMCA1) protein was used as a loading control for plasma membrane fractions. The following primary antibodies were used for Western blot analysis: anti-CaSR (ADD, ab19347, Abcam), anti-calnexin (ab2301, Millipore) and anti-PMCA1 (ab190355, Abcam). The Western blots were visualized using an Immuno-Star WesternC kit (BioRad) on a BioRad Chemidoc XRS+ system (50).

### Ca^2+^_i_ measurements

Ca^2+^_e_-induced Ca^2+^_i_ responses were measured by Fluo-4 calcium assays as previously described (52). HEK293 cells were plated in 96-well plates (Corning) and transiently transfected with 1000 ng/mL WT or mutant pEGFP-N1-CaSR constructs. Ten wells were transfected with each construct. Cells were loaded with Fluo-4 dye, prepared according to manufacturer’s instructions (Invitrogen), for 30–60 min at 37°C. Baseline measurements were made for each well and increasing doses of CaCl_2_ injected automatically into each well. One well was used for each [Ca^2+^]_e_. Changes in Ca^2+^_i_ were recorded on a PHERAstar instrument (BMG Labtech) at 37°C with an excitation filter of 485 nm and an emission filter of 520 nm. The peak mean fluorescence ratio of the transient response following each individual stimulus was measured using MARS data analysis software (BMG Labtech). Responses were normalized to the maximal response of WT expressing cells and plotted using GraphPad Prism (29). Assays were performed in 4–12 biological replicates (independently transfected wells on separate plates, performed on at least 4 different days) for each of the expression constructs. Statistical analysis was performed using two-way analysis of variance (ANOVA) and the *F* test for EC_50_ values (50,52).

### Functional assays

AlphaScreen assays were undertaken to measure pERK using cells transiently transfected with 100 ng of WT or mutant pEGFP-N1-CaSR constructs. Cells were treated with 0.1–10 mM CaCl_2_ for 5 min then lysed in Surefire lysis buffer and pERK and total ERK assays performed, as previously described (10,52). The fluorescence signal in AlphaScreen assays was measured using the PHERAstar FS microplate reader (BMG Labtech) (10,52). Assays were performed in 6–12 biological replicates (independently transfected wells, performed on at least 3 different days) for each of the expression constructs. Luciferase reporter assays were undertaken to measure SRE and NFAT responses, as previously described (50, 52). Cells were transiently transfected with 100 ng/mL of the WT or mutant pEGFP-N1-CaSR constructs, 100 ng/mL luciferase construct (either pGL4-NFAT or pGL4-SRE) and 10 ng/mL pRL. At 48 h post-transfection, cells were then treated with 0–10 mM CaCl_2_ and incubated for 4 h prior to lysis and measurement of luciferase activity using Dual-Glo Luciferase (Promega) on a Veritas Luminometer (Promega), as previously described (50). Luciferase:renilla ratios were expressed as fold-changes relative to responses at basal CaCl_2_ concentrations (0.1 mM). Assays were performed in 8–12 biological replicates (independently transfected wells, performed on at least 4 different days) for each of the expression constructs. Statistical analysis was performed using two-way ANOVA. To construct the bias plots the responses at each [Ca^2+^]_e_ in Ca^2+^_i_ (x-axis) and pERK (y-axis) assays, were plotted against each other, as previously described (29). Data was normalized to WT levels, expressed between 0 and 100%. If the agonist showed no preference for one pathway over another the concentration-response curves would be coincident, and the bias plot would overlay the line of identity (dotted line in figures) (29). If the WT or mutant CaSR couples more favourably to one pathway over another, the bias plot line will fall on either side of the line towards the preferred pathway.

### Bias factor calculation

Bias factor calculations were based on previous studies (53,54). To normalise data, responses of each mutant CaSR construct were expressed relative to WT as a percentage. Nonlinear regression of concentration-response curves was performed with GraphPad Prism (GraphPad) and the EC_50_ and E_max_ calculated. The intrinsic relative activity (RA_i_) value was calculated using the following equation for each mutant and WT CaSR:

$$ RA1=\frac{Emax.1\ EC50.2}{EC50.1\ Emax2} $$

where ‘.1’ denotes response 1 (Ca^2+^_i_) and ‘.2’ denotes response 2 (pERK). The bias factor was calculated relative to WT as follows:

$$ Bias\ factor=\mathit{log}\ \frac{RA1. mutant}{RA1. WT} $$

## Statistical analysis

A minimum of four independent biological replicates (independently transfected wells, performed on at least 3 different days) were used for all statistical comparisons. All data was analysed by two-way ANOVA with Tukey’s multiple-comparisons test. Statistical analyses were undertaken using GraphPad Prism (GraphPad), and a value of *P*-value < 0.05 was considered significant for all analyses. Comparison of EC_50_ values was performed using the *F* test (50,52).

## Supplementary Material

Supplementary Data
