# Supplementary material for: Calcium-sensing receptor residues with loss- and gain-of-function mutations are located in regions of conformational change and cause signalling bias
Source: Hum Mol Genet. 2018 Jul 20;27(21):3720–33. doi: 10.1093/hmg/ddy263 (PMC6196656; doi:10.1093/hmg/ddy263)
Supplement: Supplementary Data [file ddy263_supp.zip › HMG_SI_060718.docx]

**SUPPLEMENTARY MATERIAL**

**Calcium-sensing receptor residues with loss- and gain-of-function mutations are located in regions of conformational change and cause signalling bias**

Caroline M. Gorvin, Morten Frost, Tomas Malinauskas, Treena Cranston, Hannah Boon, Christian Siebold, E. Yvonne Jones, Fadil M. Hannan, Rajesh V. Thakker

**SUPPORTING INFORMATION**

**Figure S1 Location of CaSR ECD switch residues**

**
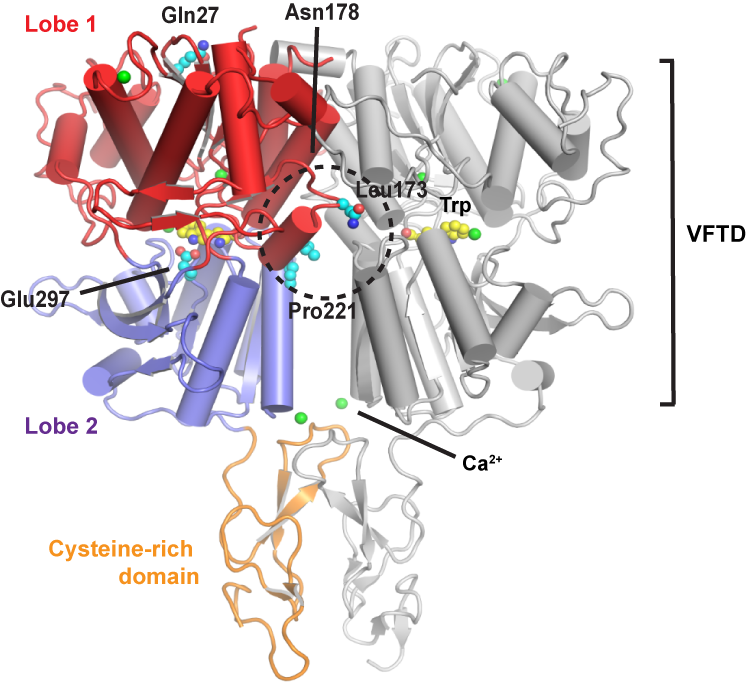
**

Crystal structure of the dimeric human CaSR extracellular domain (ECD) (PDB ID 5K5S) (1). The CaSR ECD monomer is comprised of: the venus-flytrap domain (VFTD) consisting of two lobes (lobe 1 (red) and lobe 2 (blue)), joined by a hinge region (black dashed circle) and a cysteine-rich domain (orange). Side chains of switch residues Gln27 and Asn178, and previously reported switch residues Leu173, Pro221 and Glu297 are shown as spheres (carbon, cyan; oxygen, red; nitrogen, blue). Bound calcium ions and the carbon atoms of bound tryptophan are shown as green and yellow spheres, respectively.

**Figure S2 Expression of wild-type and mutant CaSR constructs by transient transfection in HEK293 cells**

**
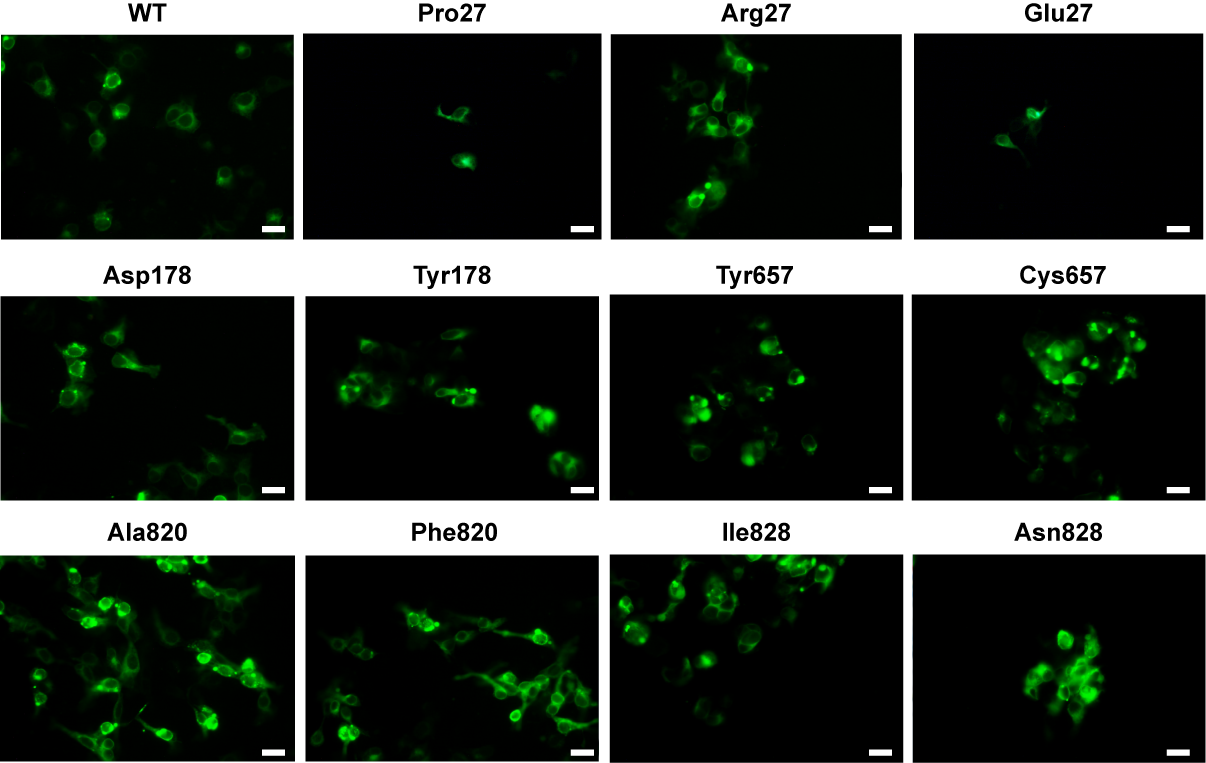
**

Fluorescence microscopy of HEK293 cells transiently transfected with WT or mutant CaSR constructs performed as a transfection control. GFP expression in these cells indicated successful transfection by these constructs. Scale bar = 10µm.

**Figure S3 Cell surface expression of mutations affecting CaSR switch residues**

**
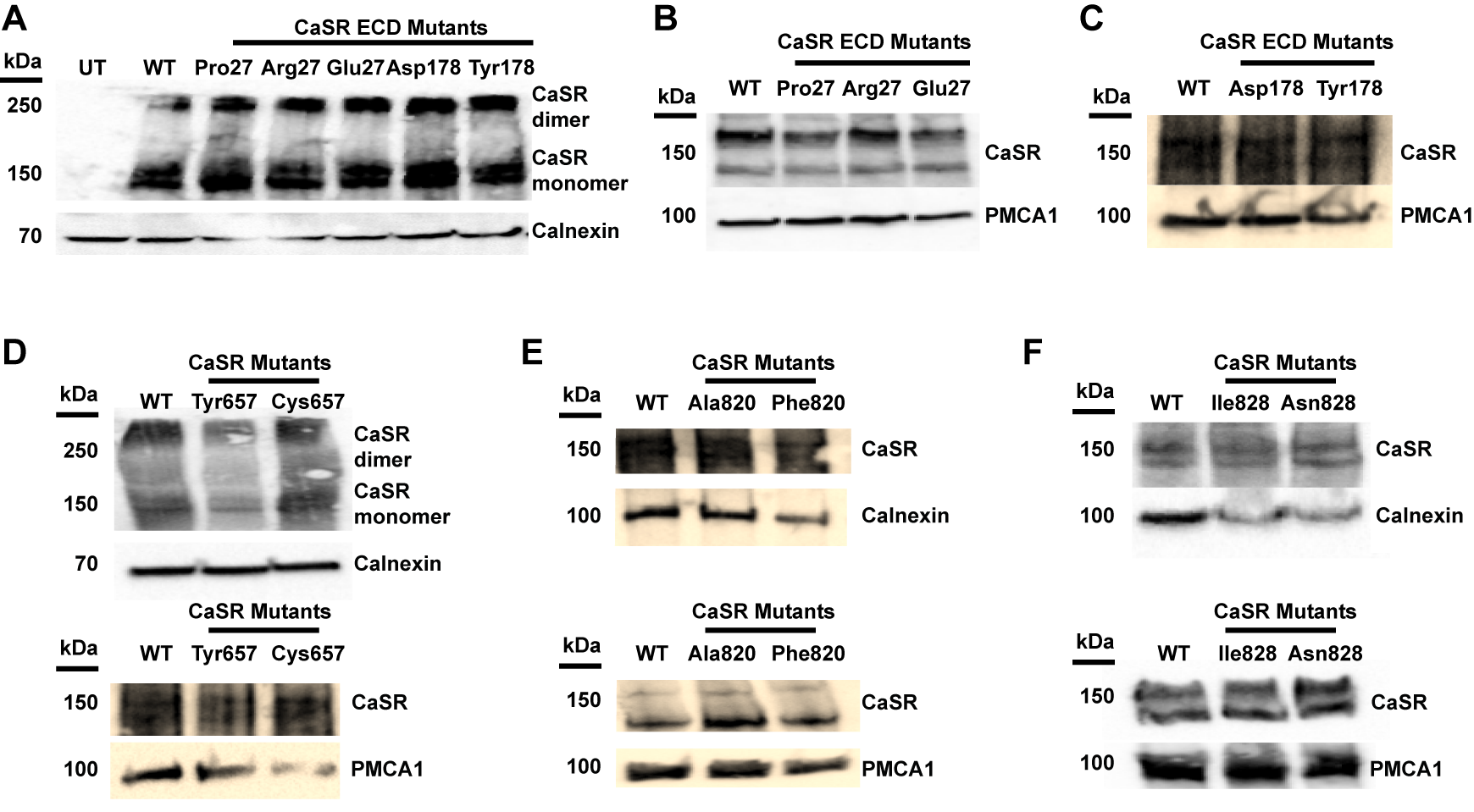
**

(**A**) Western blot analyses of total CaSR protein expression in cells transiently transfected with CaSR WT or ECD switch residue mutants. (**B-C**) Western blot analyses of CaSR in plasma membrane fractions of cells transiently transfected with CaSR WT or ECD **B**) Gln27 and (**C**) Asn178 switch residue mutants. (**D-F**) Western blot analyses of total CaSR protein (Top) or cell surface expression in plasma membrane fractions (Bottom) of cells transiently transfected with CaSR WT, or (**D**) Ser657, (**E**) Ser820, or (**F**) Thr828 switch residue mutants. Calnexin was used as a housekeeping protein for total cell lysates and the plasma membrane Ca^2+^-ATPase (PMCA1) as a housekeeping protein for plasma membrane fractions.

**Table S1 Predicted functional consequences of mutations affecting five CaSR switch residues**

| **Wild-type** |  | **FHH1 mutation** | | | | |  | | **ADH1 mutation** | | | | | |  |
| --- | --- | --- | --- | --- | --- | --- | --- | --- | --- | --- | --- | --- | --- | --- | --- |
| **Codon** |  | **Amino acid** |  | **MutationTaster^a^** |  | **Polyphen-2^b^** | |  | | **Amino acid** |  | **MutationTaster** |  | **Polyphen-2** | |
| **Gln27** |  | Pro |  | Disease-causing 0.99 |  | Probably damaging 0.99 | |  | | Glu |  | Disease-causing 0.99 |  | Possibly damaging 0.90 | |
|  |  | Arg |  | Disease-causing 0.99 |  | Possibly damaging 0.92 | |  | |  |  |  |  |  | |
| **Asn178** |  | Asp |  | Disease-causing 0.99 |  | Probably damaging 0.99 | |  | | Tyr |  | Disease-causing 0.99 |  | Probably damaging 1.00 | |
| **Ser657** |  | Tyr |  | Disease-causing 0.99 |  | Probably damaging 1.00 | |  | | Cys |  | Disease-causing 0.99 |  | Probably damaging 1.00 | |
| **Ser820** |  | Ala |  | Disease-causing 0.99 |  | Probably damaging 0.99 | |  | | Phe |  | Disease-causing 0.99 |  | Probably damaging 1.00 | |
| **Thr828** |  | Ile |  | Disease-causing 0.99 |  | Probably damaging 1.00 | |  | | Asn |  | Disease-causing 1.00 |  | Probably damaging 1.00 | |

^a^MutationTaster predicts effects of amino acid substitutions based on evolutionary conservation and loss of protein features, and gives a qualitative score of either disease-causing or benign (2). ^b^Polyphen-2 predicts effects based on sequence homology, protein databank structures, and protein family database (pfam) annotations (3), and gives a qualitative score of probably damaging, possibly damaging, benign, or unknown. Quantitative scores for both programs are based on the probability that the change is damaging, i.e. the nearer to 0 the more benign. A mutation is classified as disease-causing or probably damaging if the score is >0.85 (3).

**Table S2 EC_50_ and E_max_ values and calculated bias factors from Ca^2+^_i_ and pERK studies of five CaSR switch residues**

|  | **Ca^2+^_i_ normalised to WT** | | |  | **pERK normalised to WT** | | |  |  |
| --- | --- | --- | --- | --- | --- | --- | --- | --- | --- |
| **CaSR** | **EC_50_**  **(95% CI)** |  | **E_max_**  **(95% CI)** |  | **EC_50_**  **(95% CI)** |  | **E_max_**  **(95% CI)** |  | **Bias factor (pathway preference)** |
| **WT** | 2.68  (2.27-3.09) |  | 100  (80.54-119.46) |  | 4.48  (3.48-5.48) |  | 100  (87.23-112.77) |  | 0  (Ca^2+^_i_) |
| **Pro27** | 4.21  (3.92-4.56)** |  | 77.07  (69.11-85.11) |  | 10.78  (8.38-13.18)*** |  | 20.13  (9.78-30.48)**** |  | 0.66  (Ca^2+^_i_) |
| **Arg27** | 3.26  (2.95-3.57) |  | 81.57  (75.53-87.60) |  | 7.68  (6.61-8.02)*** |  | 84.50  (75.84-93.15) |  | 0.03  (Ca^2+^_i_) |
| **Glu27** | 3.11  (3.13-3.68) |  | 173.09  (148.72-197.47)**** |  | 4.63  (3.86-5.44) |  | 148.65  (145.30-152.00)**** |  | -0.09  (pERK) |
| **Asp178** | 4.29  (3.96-4.62)** |  | 75.35  (69.49-79.21)** |  | 5.22  (4.66-6.01) |  | 126.69  (105.15-148.23) |  | -0.36  (pERK) |
| **Tyr178** | 1.65  (1.30-2.03)* |  | 110.40  (94.13-126.67) |  | 4.51  (2.71-6.81) |  | 149.95  (124.79-175.10)**** |  | 0.05  (Ca^2+^_i_) |
| **Tyr657** | 3.81  (3.49-4.03)** |  | 47.97  (36.27-59.66)**** |  | 6.30  (4.98-7.53)* |  | 37.40  (12.45-62.35)**** |  | 0.10  (Ca^2+^_i_) |
| **Cys657** | 2.41  (2.06-2.77) |  | 159.95  (134.65-185.25)**** |  | 8.64  (7.75-9.72)*** |  | 94.37  (76.17-112.57) |  | 0.56  (Ca^2+^_i_) |
| **Ala820** | 4.68  (3.95-5.41)* |  | 74.01  (47.28-100.75) |  | 2.64  (1.10-4.18) |  | 92.24  (87.80-96.67) |  | -0.36  (pERK) |
| **Phe820** | 0.80  (0.57-1.08)** |  | 57.64  (30.33-84.94) |  | 2.58  (1.88-3.28)** |  | 53.77  (45.11-62.43)**** |  | -0.06  (pERK) |
| **Ile828** | 3.21  (2.77-3.63) |  | 70.32  (64.49-76.16)* |  | 5.26  (3.97-6.92) |  | 77.83  (62.67-92.99)* |  | 0.05  (Ca^2+^_i_) |
| **Asn828** | 2.04  (1.88-2.2.1)* |  | 110.33  (105.34-115.32) |  | 4.76  (4.17-5.39) |  | 122.55  (106.46-138.64)* |  | 0.10  (Ca^2+^_i_) |

EC_50_ and E_max_ values for Ca^2+^_i_ (assessed by fluo-4 calcium assays) and pERK (assessed by AlphaScreen assays) with 95% confidence intervals (CI). To derive the E_max_, all values were expressed as a percentage relative to the E_max_ of WT CaSR. The E_max_ and EC_50_ values from each assay were used to calculate intrinsic relative activity (RA_i_) values, and the bias factor calculated for each CaSR mutant compared to WT, as reported (4, 5). WT CaSR is biased towards Ca^2+^_i_ signalling. Mutant receptors with a positive value are similarly biased towards Ca^2+^_i_, and negative values biased towards pERK pathways. Statistical analyses for EC_50_ were performed by the F-test and for E_max_ by two-way ANOVA. ****p<0.0001, ***p<0.001, **p<0.01, *p<0.05 compared to WT CaSR.

**References**

1 Geng, Y., Mosyak, L., Kurinov, I., Zuo, H., Sturchler, E., Cheng, T.C., Subramanyam, P., Brown, A.P., Brennan, S.C., Mun, H.C. *et al.* (2016) Structural mechanism of ligand activation in human calcium-sensing receptor. *Elife*, **5**.

2 Schwarz, J.M., Rodelsperger, C., Schuelke, M. and Seelow, D. (2010) MutationTaster evaluates disease-causing potential of sequence alterations. *Nat Methods*, **7**, 575-576.

3 Adzhubei, I.A., Schmidt, S., Peshkin, L., Ramensky, V.E., Gerasimova, A., Bork, P., Kondrashov, A.S. and Sunyaev, S.R. (2010) A method and server for predicting damaging missense mutations. *Nat Methods*, **7**, 248-249.

4 Gundry, J., Glenn, R., Alagesan, P. and Rajagopal, S. (2017) A Practical Guide to Approaching Biased Agonism at G Protein Coupled Receptors. *Front Neurosci*, **11**, 17.

5 Luttrell, L.M., Maudsley, S. and Bohn, L.M. (2015) Fulfilling the Promise of "Biased" G Protein-Coupled Receptor Agonism. *Mol Pharmacol*, **88**, 579-588.
